# Face Manipulation Detection Based on Supervised Multi-Feature Fusion Attention Network

**DOI:** 10.3390/s21248181

**Published:** 2021-12-08

**Authors:** Lin Cao, Wenjun Sheng, Fan Zhang, Kangning Du, Chong Fu, Peiran Song

**Affiliations:** 1 The Key Laboratory of the Ministry of Education for Optoelectronic Measurement Technology and Instrument, Beijing Information Science and Technology University, Beijing 100101, China; CharLin26@163.com (L.C.); qiaoba1995@163.com (W.S.); kangningdu@outlook.com (K.D.); peiransong@bistu.edu.cn (P.S.); 2 School of Computer Science and Engineering, Northeastern University, Shenyang 110169, China; fuchong@mail.neu.edu.cn

**Keywords:** face manipulation detection, capsule network, attention mechanism, supervised multi-feature fusion attention network

## Abstract

Nowadays, faces in videos can be easily replaced with the development of deep learning, and these manipulated videos are realistic and cannot be distinguished by human eyes. Some people maliciously use the technology to attack others, especially celebrities and politicians, causing destructive social impacts. Therefore, it is imperative to design an accurate method for detecting face manipulation. However, most of the existing methods adopt single convolutional neural network as the feature extraction module, causing the extracted features to be inconsistent with the human visual mechanism. Moreover, the rich details and semantic information cannot be reflected with single feature, limiting the detection performance. Therefore, this paper tackles the above problems by proposing a novel face manipulation detection method based on a supervised multi-feature fusion attention network (SMFAN). Specifically, the capsule network is used for face manipulation detection, and the SMFAN is added to the original capsule network to extract details of the fake face image. Further, the focal loss is used to realize hard example mining. Finally, the experimental results on the public dataset FaceForensics++ show that the proposed method has better performance.

## 1. Introduction

Face manipulation technology is a novel way to replace human faces in videos. Now, the realization of face manipulation is getting easier, benefiting from the development of convolutional neural networks (CNN) [1] and generative adversarial nets (GANs) [2]. Due to the entertainment and simplicity, it becomes increasingly popular. Nowadays, face manipulation methods can be divided into two categories: face synthesis and face swap.

**Figure 1:**
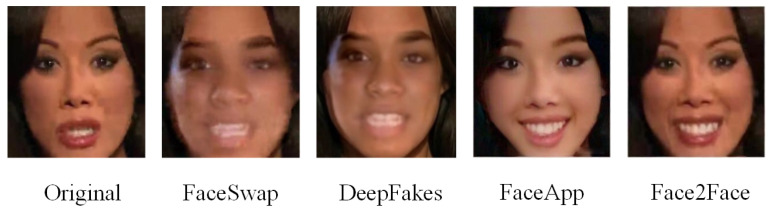
Face manipulated images.

Face synthesis mainly generates non-existent but incredibly realistic human faces through GAN (such as Cycle-Consistent Adversarial Networks (CycleGAN)  [3] and Star Generative Adversarial Nets (StarGAN) [4]). In contrast, face swap replaces one person’s face with another person’s face, usually through two methods: FaceSwap method based on computer graphics and Deepfake method based on deep learning. Moreover, face expression swap, whose popular ways are Face2Face and FaceApp, modifies facial expressions, such as changing the crying expression into the laugh. As shown in Figure 1, among all four face images, only the first one is a natural optical image, and the remaining images are manipulated by various methods. Nevertheless, most manipulated faces are not detected due to the poor privacy of faces and the advanced manipulation technology. Using face manipulation technology to make fake videos will undoubtedly ruin the image of celebrities and politicians, for instance, the manipulated video of U.S. democratic leader Nancy Pelosi getting drunk that Trump shared on Facebook aroused great attention on the Internet. In addition, Obama’s video was also used to make manipulated videos. The manipulated targets are not only worldwide officials but also actors, singers, and even ordinary people. Therefore, it is necessary to design a method to detect all the manipulation types.

When advanced face manipulation methods appear, various face manipulation detection methods are also emerging. Methods based on CNN [1] and some of its variants [5] have made significant progress. These methods can easily handle high-dimensional information and reduce the complexity of the network model. Recurrent Neural Network (RNN) [6,7], especially the Long Short-Term Memory (LSTM), also achieves good performance within the field. Besides, some technologies in other fields, such as natural language processing (NLP) [8], can also be used in the face manipulation detection task. Furthermore, the development of capsule network [9] has brought new way for face manipulation detection task. However, the above-mentioned methods only use single CNN as the feature extraction module, especially the CNN used in the capsule network method [9] only has three layers, which results in poor consistency between the features and the human visual mechanism. Therefore, the proposed method tackles above problems by proposing face manipulation detection method based on the supervised multi-feature fusion attention network named SMFAN.

The proposed model has apparent strengths. In particular, based on the original capsule network, the proposed model predicts the attention map by SMFAN. Then, in order to improve the problem of insufficient detail feature extracted by the original capsule network, the attention map is sent to the feature extraction module of the original capsule network. In addition, the introduction of focal loss [10] is utilized to prevent the vast number of easy negatives from overwhelming the detector during training. Finally, the experiments evaluate the proposed model on the FaceForensics++ [11] dataset, and the experimental results show that the proposed model is better than the original capsule network.

In summary, the main contributions of this paper are twofold:

- Based on the work of the capsule network [9], the SMFAN is introduced to optimize the feature extraction module.
- Use focal loss to down-weight the loss assigned to the well-classified examples.

The remainder of this paper is organized as follows. Section 2  introduces related work. Section 3  describes the proposed method, including the overall network structure, the SMFAN structure, and the loss function. Section 4  shows the experimental results, and Section 5  summarizes this paper.

## 2. Related Works

### 2.1. Face Manipulation Methods

#### 2.1.1. Face Synthesis

Face synthesis tends to generate non-existent but incredibly realistic human faces. CycleGAN [3] is a suitable means to do the task. Through introducing the adversarial loss [2] and cycle consistency loss, CycleGAN  [3] addresses the problem of unpaired training data of the image-to-image translation in the process of synthesis, achieving a good performance within the field of face synthesis. Other GAN, such as StarGAN [4] and Style Generative Adversarial Nets (StyleGAN) [12], can also synthesize realistic images.

#### 2.1.2. Face Swap

Now, FaceSwap is a mature software to replace faces in videos, which is achieved by two steps: extraction and training. Deepfake, whose name is combined by deep learning and fake photos, is another way to realize face swap. The technology emerges from encoder–decoder structure and is updated by GAN such as Face Swapping Generative Adversarial Nets (FSGAN) [13] and Relativistic Standard Generative Adversarial Nets (RSGAN) [14], which contains three steps: face positioning, face conversion, and image stitching.

Among face expression swap methods, Face2Face [15] is an advanced way. The implementation of Face2Face can be divided into the following steps. The first step is to reconstruct the shape identity of the target actor and track both the expressions of the source and target actor’s video. Next, use transfer functions to transfer expressions from the source to the target actor in real-time. Then, re-render the target’s face and composite it with the target video’s background. Through generating a realistic mouth by the synthesis approach, a face generated by Face2Face is finally obtained.

### 2.2. Face Manipulation Detection Methods

#### 2.2.1. Detection Methods Based on Convolutional Features

CNN is a good tool for extracting features. Rossler et al. [11] proposed a detection method based on CNN, which extracts features based on XceptionNet [16] and then sends the features into the classifier to detect manipulated faces. Dang et al. [17] introduced the attention mechanism based on salient features of face, where a regression-based method was used to estimate the attention map and then multiplied it with the input feature maps to optimize. In addition, attention loss was introduced into the model, which could be got by pairing the fake image with its corresponding source image. Typically, the absolute value of the difference was calculated in the RGB channel pixel by pixel, next converting it to grayscale, then normalizing it, and finally calculating the 1-norm of the obtained value. Durall et al. [18] found that most of the existing face manipulation methods may change the spectral characteristics of the image, thereby causing high-frequency distortion. Therefore, different from others, features of frequency domain instead of time domain were used as input into the CNN. After applying the discrete Fourier transform to the image, the azimuth angle on the radial frequency was integrated to obtain the spectral characteristics of the image. Finally, the support vector machine (SVM) was employed for classification. Guo et al. [19] denied the above methods based on CNN. They believed that in the field of face manipulation detection, CNN should not learn the image’s content but the manipulation traces. Therefore, the work in [19] improved the constrained convolutional layer [5] and proposed an adaptive residual extraction network, which could be utilized as image preprocessing to achieve the purpose of suppressing image content. Deressa et al. [8] made use of Transformer for computer vision tasks, proposing a generalized convolutional vision transformer architecture to achieve the face manipulation detection. However, the above-mentioned methods enormously depend on powerful CNN and are short of robustness.

#### 2.2.2. Detection Methods Based on Image and Temporal Features

Considering that video coherence is not considered in the existing face manipulation detection methods, Sabir et al. [7] utilized RNN to train an end-to-end model. Furthermore, similar to the work in [7], Guera et al. [6] proposed to use LSTM to compare the difference between frames. The method input the feature maps extracted by CNN into the LSTM, which broke out the common methods based on image detection. These methods greatly rely on powerful CNN, and thus the similar problems are suffered as methods based on convolutional features.

#### 2.2.3. Detection Methods Based on Steganalysis Features

Face manipulation detection is essential to judge whether there is secret information in human faces, so steganalysis is widely employed. Zhou et al. [20] proposed a two-stream network for face manipulation detection, where the streams were classification stream and patch triplet stream, respectively. Among them, GoogLeNet [21] was adopted for face manipulation classifier, and the patch triplet stream based on patch-level steganalysis features [22] was adopted for capturing low-level camera characteristics and local noise residuals. Inspired by steganalysis and natural image statistics, Nataraj et al. [23] employed the co-occurrence matrix to identify the confidential data in the image, and the detection of manipulated images and the location of the manipulated parts were finally realized. However, the problem of difficult feature extraction in steganalysis is difficult to solve.

For improving the robustness, Nguyen et al. [9] proposed a detection method based on capsule network. Compared with the traditional CNN, the capsule network can reduce the error caused by the change of the posture information, such as the direction and angle of the face. Therefore, they used the capsule network for face manipulation detection. However, compared with the current detection methods based on CNN, the feature extraction module of the capsule network is too shallow, causing the powerful feature extraction capabilities of CNN to be lost. Aiming at the existing problems of the capsule network, this paper proposes SMFAN to detect manipulated faces.

#### 2.2.4. Multi-Feature Fusion Methods

   Recently, multi-feature fusion methods shine in deep learning. Wang et al. [24] proposed a multimodal gait feature fusion identity multimodal gait information algorithm, which has achieved superior performance in the field of identity-recognition. Zhao et al. [25] used the technology of aggregating the nodes of graphs in the face clustering and achieved success. Liu et al. [26] combined the attention mechanism with bidirectional gated recurrent units to improve the interpretability in the field of point-of-interest prediction model. Yang et al. [27] applied the mechanism of multi-level fusion to named entity recognition, which worked well in natural language processing. Qin et al. [28] proposed feature fusion attention network for single image dehazing, surpassing previous state-of-the-art single image dehazing methods.

## 3. Face Manipulation Detection Based on Supervised Multi-Feature Fusion Attention Network

### 3.1. The Overall Framework

**Figure 2:**
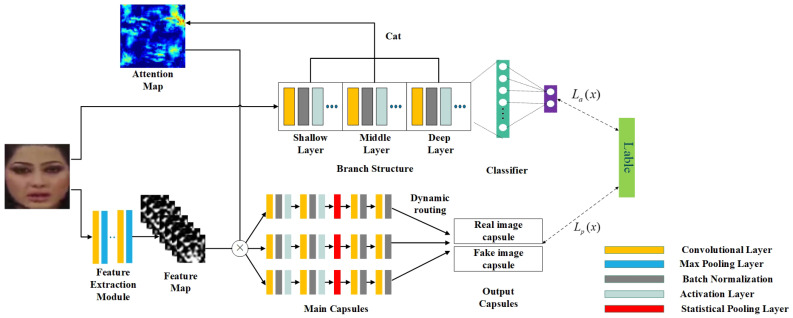
Network structure.

Traditional CNN employs scalars to represent neurons and weights, then estimates the probability of various features. In this paper, the capsule network is employed to predict the probability of feature with vector norm, which effectively reduces the error caused by the change of face posture information, such as the change of direction and angle during the prediction process. As shown in the lower part in Figure 2, the input images pass through the feature extraction module to obtain the feature maps. Then, they are input into the capsule network, which is divided into three main capsules and two output capsules. After the main capsules process the input features, the corresponding outputs are sent to two output capsules through the dynamic routing algorithm [9].

However, the original capsule network has obvious defects. It only uses the first three layers of VGG19 [29] as the feature extraction module, which weakens the detection accuracy. In order to optimize the feature extraction module in the original capsule network, the upper part of Figure 2, namely, SMFAN, is introduced. Specifically, the face is first input into the SMFAN to extract features. Then, the features are input into the classifier, which are divided into two categories: natural or manipulated face. Moreover, the result of the classifier is compared with the correct label of the face. At the same time, the process will output an attention map which is used for optimizing the feature extraction module of the capsule network. With the continuous optimization of parameters during training process, the output attention map is more and more in line with the human visual mechanism, and the optimization result of the feature extraction module is getting better and better. In addition, focal loss, which is proposed to address examples imbalance by reshaping the standard cross entropy loss, is adopted to supervise the training of model to improve detection accuracy.

### 3.2. Supervised Multi-Feature Fusion Attention Network

The original capsule network makes up for the shortcomings of traditional CNN to a certain extent. However, its feature extraction module is too shallow. As a result, the insufficient details of extracted features are input into the main capsules, which limits detection accuracy.

In recent years, the attention mechanism has become an essential concept in deep learning, especially computer vision. The attention mechanism that conforms to the human visual mechanism can perform intuitive visual interpretation for images or videos. The attention structure can be straightforward, just like directly introducing a convolutional layer and activation function into the original network and multiplying the obtained attention map with the original feature map. At present, the attention mechanism in many works is a variant of the above mode, that is, operating on the convolutional layer and changing the way to obtain the attention maps. For example, Sitaula et al. [30] concatenated the image after maximum pooling and average pooling, and then multiplied it with the feature map of the original VGG-16 after convolution. Dang et al. [17] introduced the attention mechanism based on salient features of face, where a regression-based method was used to estimate the attention map and then multiplied it with the input feature maps to optimize. However, the attention maps obtained by the above methods are all unsupervised, so the perception of image details is poor. Therefore, this paper proposes the SMFAN, which is a separate network to estimate the attention map. Compared with the traditional attention network, the proposed attention network has supervision information, and the purpose is to estimate the attention map that is in line with the human visual mechanism. The attention map contains low-level, middle-level, and high-level features, which has detailed information owned by the shallow layer and semantic information owned by the deep layer. Furthermore, it will be continuously optimized during training process. Finally, the obtained attention map is introduced into the feature extraction module of the capsule network to optimize detailed features while retaining the structure of the capsule network.

**Figure 3:**
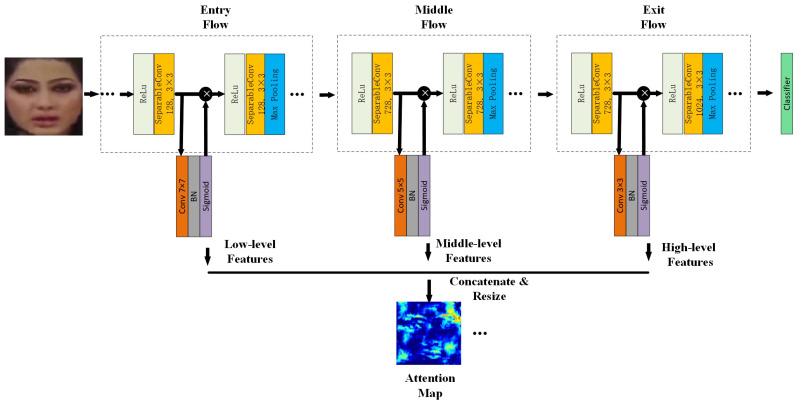
Supervised attention network structure.

The process of obtaining the attention map is shown in Figure 3. XceptionNet, pre-trained by Imagenet  [31], is adopted as the attention network. The reason why XceptionNet is used is that it serves as the baseline network in face manipulation detection, which is also one of the best performing CNNs within this field. In addition, we conducted comparative experiments in Section 4.2.4 to prove that XceptionNet is a better choice.

Estimating the attention map is realized by a convolutional layer, batch normalization (BN), and sigmoid activation function. Specifically, the attention map is estimated after the first separable convolution of the entry stream, the middle stream, and the exit stream, respectively, and the size of the convolution kernel is 7×7, 5×5, and 3×3, respectively. Then, we resize the three attention maps to 64×64 and concatenate them. Finally, after changing the dimension of the cascading attention maps, the multi-feature fusion attention map is obtained. The obtained attention map is multiplied with the feature maps extracted from the feature extraction module to enhance the representability ability. The optimized feature map is defined by Equation (1):

(1) $$A^{\prime }\left(x\right)=\left(1+Con\left(M_{L}\left(x\right),M_{M}\left(x\right),M_{H}\left(x\right)\right)\right)\cdot A\left(x\right),$$

where $M_{L}\left(x\right)$ is the low-level features, $M_{M}\left(x\right)$ is the middle-level features, $M_{H}\left(x\right)$ is the high-level features, Con represents the concatenation operation, and A(x) is the feature maps of the feature extraction module.

**Figure 4:**
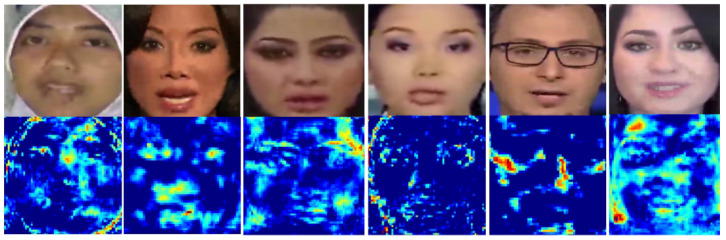
Visualization of attention map.

It can be seen from Equation (1) that each pixel of the estimated attention map is added with a constant 1. The purpose is that the constant can highlight the feature maps at the peak of the attention map and prevent the low pixel values from falling to 0. Experiments in Section 4.2.4 will evaluate the effect of adding the constant 1. As shown in Figure 4, some attention maps are visualized. The first row is the cropped face images, and the second row is the attention maps corresponding to the face images of the first row. It can be seen that the attention mechanism can effectively pay attention to the critical parts of the face, such as the eyes, nose, mouth, and the face profile.

### 3.3. Loss Function

Traditional cross-entropy loss treats all examples equally and is utilized by most of the current methods, which causes that these methods to treat hard examples like easy examples. Using models trained by the cross-entropy loss, unpredictable errors may occur, especially in detecting hard examples.

Focal loss [10] based on cross-entropy loss, by introducing a weighting factor α and a modulating factor γ, addresses the scenario where there is an extreme imbalance between positive and negative classes during training. The loss reduces the weights of easy examples in training, so it can also be understood as a kind of hard example mining. Therefore, focal loss is adopted to improve the detection performance of hard examples in this paper, and the loss function *L* is defined by Equation (2):

(2) $$L{=\{}_{-\left(1-\alpha \right){\left(y^{\prime }\right)}^{\gamma }\mathrm{lg}\left(1-y^{\prime }\right),y=0}^{-\alpha {\left(1-y^{\prime }\right)}^{\gamma }\mathrm{lg}y^{\prime },\;\;\;\;\;\;\;\;\;\;\;\;\;\;\;\;\;\;\;\;\;\;\;y=1}\;,$$

where $y^{\prime }$ is the output, α is used to balance the imbalance in the number of positive and negative examples, γ is used to reduce the loss contribution from easy examples and extends the range in which an example receives low loss, and *y* is the label, where y=1 represents positive examples and y=0 represents negative examples.

The loss is used in the capsule network and the SMFAN, and the total loss L(x) in the training process is shown in Equation (3):

(3) $$L\left(x\right)=L_{a}\left(x\right)+L_{p}\left(x\right),$$

where $L_{a}\left(x\right)$ is the focal loss of the SMFAN branch, and $L_{p}\left(x\right)$ is the focal loss of the capsule network. The definitions of the above two losses are defined by Equations (4) and (5):

(4) $$L_{a}\left(x\right){=\{}_{-\left(1-\alpha_{a}\right){\left({y^{\prime }}_{a}\right)}^{\gamma_{a}}\left(1-{y_{a}}^{\prime }\right),y=0}^{-\alpha_{a}{\left(1-{y^{\prime }}_{a}\right)}^{\gamma_{a}}\mathrm{lg}{y^{\prime }}_{a},\;\;\;\;\;\;\;\;\;\;\;\;\;\;\;\;y=1},$$

(5) $$L_{p}\left(x\right){=\{}_{-\left(1-\alpha_{p}\right){\left({y^{\prime }}_{p}\right)}^{\gamma_{p}}\mathrm{lg}\left(1-{y_{p}}^{\prime }\right),y=0}^{-\alpha_{p}{\left(1-{y^{\prime }}_{p}\right)}^{\gamma_{p}}\mathrm{lg}{y^{\prime }}_{p},\;\;\;\;\;\;\;\;\;\;\;\;\;\;\;\;y=1},$$

where ${y^{\prime }}_{a}$ and ${y^{\prime }}_{p}$ are the output of the SMFAN branch and capsule network, respectively. $\alpha_{a}$ and $\alpha_{p}$ are used to balance the importance of positive and negative examples in the SMFAN branch and capsule network, respectively. $\gamma_{a}$ and $\gamma_{p}$ are used to reduce the loss contribution from well-classified examples in the SMFAN branch and capsule network, respectively. *y* is the label.

In summary, the process of the SMFAN algorithm is shown in Algorithm 1.
**Algorithm 1** SMFAN **Input:** The image *I*; **Output:** The label: 0 (manipulated) or 1 (real); **for all** training images **do**  1. Input the image *I* to the Xception network for classification (real or manipulated);  2. Estimate the attention maps $M_{L}\left(x\right)$, $M_{M}\left(x\right)$, $M_{H}\left(x\right)$ after the first separable convolution of the entry stream, the middle stream, and the exit stream;  3. Resize the three attention maps to 64 × 64 and concatenate them to obtain the multi-feature fusion attention map $Con(M_{L}\left(x\right),M_{M}\left(x\right),M_{H}\left(x\right))$;  4. Obtain the feature maps A(x) via the feature extraction module of the capsule network;  5. The multi-feature fusion attention map is multiplied with A(x), namely $A^{\prime }\left(x\right)=\left(1+Con\left(M_{L}\left(x\right),M_{M}\left(x\right),M_{H}\left(x\right)\right)\right)\cdot A\left(x\right)$ is obtained;  6. Through the dynamic routing algorithm of the capsule network, the results are output to two output capsules, 0 or 1 respectively;  7. Calculate the total focal loss *L* and back propagate to update network parameters. **end for**

## 4. Experimental Results and Analysis

### 4.1. Experimental Settings

#### 4.1.1. Datasets

FaceForensics [32] dataset is used in this paper, which includes more than 1000 videos (contains 500,000 frames of images) manipulated by Face2Face. Subsequently, an extended version of the FaceForensics dataset named FaceForensics++ is released, which is further expanded by DeepFakes and FaceSwap technology. It includes 3000 fake videos (contains 1.5 million frames of images). Nowadays, it has become the benchmark dataset for most researchers in the field. Besides, Deepfake Detection Challenge (DFDC) has been released as part of the DFDC Kaggle competition, and Diverse Fake Face Dataset (DFFD) [17], including 2.6 million images, comprises various categories of face manipulations.

In this paper, three manipulation types are picked from the FaceForensics++ [11] dataset for experiments: DeepFakes, FaceSwap, and Face2Face. Among the 1000 videos of each type, 720 videos are randomly selected as the training set, and 270 frames are extracted from each video; 140 videos are randomly selected as the validation set, and the remaining 140 videos are selected as the test set. One-hundred frames are extracted from both the validation set and the test set. Afterwards, the MTCNN [33] algorithm is used to locate and recognize the faces in videos and crop the faces, which are used as the data in the experiment.

#### 4.1.2. Experimental Settings

During the training process, the input image size is 256×256. Epoch is set to 20 for training, and the batch size is set to 32. The learning rate of capsule network is 0.001, while the learning rate of the SMFAN branch is 0.0001. The Stochastic Gradient Descent (SGD) method is used for optimization. The focal losses in capsule network and the SMFAN branch are consistent, where the weighting factor $\alpha_{a}$ and $\alpha_{p}$ are 0.25, and the modulating factor $\gamma_{a}$ and $\gamma_{p}$ are 2. The Pytorch is used as the basic framework, and the GPU is NVIDIA RTX 2080Ti.

### 4.2. Experimental Results and Analysis

Based on capsule network, this paper proposes to use SMFAN to optimize the feature extraction module of capsule network, and use focal loss to replace the cross-entropy loss in the original network. In order to verify the superiority of the proposed method, the following experiments are done. Typically, all performances are reported on the same dataset and parameters, and all results are obtained based on the compressed version (compression rate is 23%) of FaceForensics++ dataset. Accuracy is used to measure the performance, which is defined by Equation (6):

(6) $$accuracy=\frac{TP+TN}{total},$$

where TP represents the number of samples that the positive class is judged to be positive, TN represents the number of samples that the negative class is judged to be negative, and total represents the total number of samples.

#### 4.2.1. Comparative Experiment

In order to verify the effectiveness of the proposed method, the proposed method is compared with the original capsule network [9]. The reason why we only compare with capsule network is that SMFAN is proposed based on the capsule network [9], which aims to improve the feature extraction ability of the original capsule network.

Table 1 shows the experimental results on DeepFakes, FaceSwap, and Face2Face. The results show that for all manipulation types, the uncertainty has a certain degree of decline, which shows that our method is more stable. In addition, the results also show that for DeepFakes and Face2Face, the accuracy of the proposed method has been improved significantly, which has increased by more than 1%. On the other hand, for the FaceSwap, the accuracy improvement is less, ~0.6%. The reasons why the increase is small include the following aspects: (1) the FaceSwap is more mature in dealing with facial jitter and (2) the pose information of the face is more realistic in the process of replacing the entire face, which weakens the advantages of the capsule network.

In addition, to verify the generalization of the proposed method, all videos of the three manipulation types are integrated together as the new training set and test set. The results are shown in Table 2. The results show that compared with the original capsule network, the proposed method’s accuracy is increased by more than 4% and the generalization ability is better. Furthermore, the performance advantage of uncertainty is obvious, which also proves its generalization and stability. The fundamental reason for the improvement lies in the important role of the attention branch, which can focus on key parts of the human face.

#### 4.2.2. Model Performance Comparison Experiment

Nowadays, state-of-the-art methods with excellent detection results have been proposed. In order to verify the superiority of the proposed model, the comparative experiment is conducted on the Face2Face manipulation type. Table 3 shows the comparison of accuracy between the proposed method and other state-of-the-art methods on the Face2Face manipulation type.

It can be seen from Table 3 that compared with state-of-the-art methods, the detection performance of the proposed method has significant improvement. Among them, the advantages obtained by the proposed method are much greater than the one proposed by Cozzolino [34] and Bayar et al. [35], there is a significant performance improvement, which is approximately 19% and 12% respectively. Compared with the methods proposed by Afchar et al. [36] and Raghavendra et al. [37], it increases by approximately 5%. Compared with the baseline method proposed by Rossler et al. [11], the accuracy has an increase of ~0.9%. Compared with the methods proposed by Li et al. [38] and Zhu et al. [39] in 2020, the proposed method is also better. In summary, because the proposed method integrates the advantages of XceptionNet in feature extraction, the rich details and semantic information brought by multi-features fusion and the robustness of the capsule network, the proposed method is better than the state-of-the-art detection methods.

#### 4.2.3. Ablation Experiment

In order to prove the respective effects of SMFAN and focal loss, the following ablation experiments are carried out: (1) introduce SMFAN and calculate its accuracy on the DeepFakes, FaceSwap, Face2Face manipulation types after training the model (replace the loss in Figure 2 with the cross-entropy loss), and (2) do not use SMFAN, only focal loss is used instead of cross-entropy loss (only use the lower part of Figure 2). The experimental results are shown in Table 4. It can be seen from Table 4 that for the manipulated types, the introduction of SMFAN greatly improves the performance, especially the DeepFakes type, which increases by approximately 0.9%. The focal loss makes the model focus on training a sparse set of hard examples, which also plays a role that cannot be ignored. In addition, for the FaceSwap type, the improvement of introducing SMFAN and focal loss is smaller than that of the DeepFakes and Face2Face types, which verifies the small improvement in the detection performance of the FaceSwap type in Section 4.2.1.

Then, the above experiments are performed again using the integrated dataset with all three types, and the experimental results are shown in Table 5. It can be seen from Table 5 that the improvement is approximately 2.7% when introduces SMFAN and 1.2% when introduces focal loss. The results also prove the above conclusion.

#### 4.2.4. Branch Structure Model Experiment

Compared with the traditional attention mechanism, the attention branch in this paper has obvious characteristics—it has supervised information and contains rich features. Through directly obtaining the attention map based on the feature extraction module of the original capsule network and multiplying it with the feature map, this paper proves that SMFAN is more reasonable. The experimental results are shown in Table 6. Compared with directly introducing attention structure, our proposed SMFAN branch is more in line with the human visual mechanism because of the supervised multi-feature fusion attention map. It can be seen from Table 6 that the detection performance of the supervised SMFAN branch is improved by ~2.4%, which is significantly better than the traditional attention structure.

In addition, the attention branches with a single feature and two-layer features (low-level and high-level) fusion are compared with the proposed three-layer features fusion (low-level, middle-level, and high-level). The experimental results are shown in Table 7. Due to equipment limitations, this paper only carries out three-layer feature fusion. It can be concluded that the more fused features, the better the performance.

Further, in order to verify the influence of the constant 1 mentioned in Section 3.2, we compare the results of whether to introduce the constant 1 in SMFAN. From the results of Table 8, the constant 1 improves the model’s accuracy, increasing by 0.28%. It can be seen that the constant 1 does have the significance in highlighting the feature maps and preventing low-value pixels.

Furthermore, we conducted comparative experiments in Table 9 to prove XceptionNet performs better than VGG-16 as the branch network. More specifically, VGG-16 has three fully connected layers, which means that VGG-16 has more parameters, and the real-time performance is poor. Moreover, the accuracy of XceptionNet is higher, so XceptionNet is used in our work.

#### 4.2.5. Time Performance Comparison Experiment

We compare the number of network parameters and processing time of different methods. The results are shown in Table 10. It can be concluded that compared with the method based on capsule network, the parameters of our method are increased by approximately 3 times, but the processing time for a single image only increased by approximately 0.5 times. When compared with the method proposed by Rossler et al., the number of parameters has increased by 7000K, but the processing time for a single image is similar. In summary, although the number of parameters of the proposed method has increased, the processing time is only slightly increased with high accuracy. In a word, the proposed method has a better balance between performance and time.

Therefore, in the scenarios of the strict real-time requirements, the proposed method may not meet the requirements because it uses time in exchange for performance. The best solution to this problem is of course to use a network with fewer parameters and higher performance, which will be one of the focuses of the future work.

## 5. Conclusions

This paper proposes a capsule network structure based on SMFAN to detect whether videos or images have been manipulated. For the input image, this paper uses SMFAN to estimate the attention map, which is used to optimize the feature extraction module of capsule network. Then capsule network is used to detect whether the input is real or fake, and the focal loss is used to improve the detection accuracy. The experimental results show that the proposed method has a significant performance improvement compared with the state-of-the-art methods, and has better discriminant ability when various manipulation types are mixed. The focus of future work will be on the real-time performance improvement. In addition, the use of improved capsule network is also a major focus of future work.

## Figures and Tables

**Table 1:** Comparison of the accuracy and the uncertainty of the proposed method and the original capsule network on different manipulation types.

Manipulation Type	Capsule Network [9]	Ours
DeepFakes	96.41 ± 2.01%	97.77 ± 1.47% (+1.36%)
FaceSwap	97.63 ± 2.14%	98.21 ± 1.13% (+0.58%)
Face2Face	97.85 ± 1.79%	99.06 ± 0.72% (+1.21%)

**Table 2:** Comparison of the accuracy and the uncertainty of the proposed method and the original capsule network on the FaceForensics++ dataset.

Dataset	Capsule Network [9]	Ours
FaceForensics++	91.80 ± 4.21%	97.23 ± 2.83% (+5.43%)

**Table 3:** Comparison of accuracy between the proposed method and state-of-the-art methods on the Face2Face manipulation type.

Detection Method	Accuracy
Cozzolino et al. [34]	79.80% (+19.26%)
Bayar et al. [35]	86.10% (+12.96%)
Afchar et al. [36]	93.40% (+5.66%)
Raghavendra et al. [37]	93.50% (+5.56%)
Li et al. [38]	97.73% (+1.33%)
Nguyen et al. [9]	97.85% (+1.21%)
Rossler et al. [11]	98.13% (+0.93%)
Zhu et al. [39]	98.22% (+2.84%)
Ours	99.06%

**Table 4:** Comparison of accuracy of introducing the SMFAN branch and focal loss on different manipulation types.

Manipulation Type	Capsule Network [9]	Add the SMFAN Branch	Add Focal Loss	Add the SMFAN Branch
DeepFakes	96.41%	97.27% (+0.86%)	96.83% (+0.52%)	97.77% (+1.36%)
FaceSwap	97.63%	97.95% (+0.32%)	97.72% (+0.09%)	98.21% (+0.58%)
Face2Face	97.85%	98.48% (+0.63%)	98.14% (+0.29%)	99.06% (+1.21%)

**Table 5:** Comparison of accuracy of introducing the SMFAN branch and focal loss on the FaceForensics++ dataset.

Dataset	Capsule Network [9]	Add the SMFAN Branch	Add Focal Loss	Add the SMFAN Branch
FaceForensics++	91.80%	94.53% (+2.73%)	92.97% (+1.17%)	97.23% (+5.43%)

**Table 6:** Comparison of accuracy of directly introducing attention and using of SMFAN branch on the FaceForensics++ dataset.

Dataset	Traditional Attention Structure	SMFAN Branch
FaceForensics++	92.76%	95.13% (+3.33%)

**Table 7:** Comparison of accuracy of features fusion of different layers on the FaceForensics++ dataset.

Dataset	One Feature	Two Features Fusion	Three Features Fusion
FaceForensics++	95.70%	96.16% (+0.91%)	97.23% (+1.53%)

**Table 8:** Comparison of accuracy of whether to introduce the constant 1 in the SMFAN branch.

Dataset	without Constant 1	Introduce Constant 1
FaceForensics++	94.85%	95.13% (+0.28%)

**Table 9:** Comparison of the accuracy of XceptionNet and VGG-16 on the FaceForensics++ dataset.

	VGG-16	XceptionNet
Number of network parameters	138,000 K	22,500 K
Time to process a single image	0.0514 s	0.0308 s
Accuracy	96.85%	97.23%

**Table 10:** Comparison of the number of network parameters and processing time between different methods.

Detection Method	Number of Network Parameters	Time to Process a Single Image
Nguyen et al. [9]	6800 K	0.0216 s
Rossler et al. [11]	22,500 K	0.0308 s
Ours	29,710 K	0.0324 s

## Data Availability

The original datasets are publicly available from Github (https://github.com/ondyari/FaceForensics).
